# Present State of Organic Chemistry

**Published:** 1838

**Authors:** M. Dumas


Art. V.—*Extracts from a memeoir on the present state of organic chemistry, read before the Royal Academy of Sciences.* By M. Dumas.

(Tanslated for the Western Journal, by J. A. W )

Mineral chemistry embraces the history of elementary bodies, of the Cinary and saline combinations. But elementary bodies may be divided into several very natural groups, so that if we study attentively the properties of any species, we may almost foresee and describe those of the species with which it is connected. The study of oxygen teaches us the history of sulphur, that of chlorine initiates us into the intimate details of the properties of iodine, &c. This task, which would appear superhuman, embracing the analysis of a thousand various substances, has nevertheless been so nearly accomplished in less than half a century, that there is now, only here and there a link to be supplied.

It may be readily conceived, that with the fifty-four elements now recognized, with a small number of combinations and with all the Cinary and saline compounds, we cannot only form all the compounds known in the mineral kingdom, but, also, an immense number of analogous substances. How shall we apply these principles, with equal success, to organic chemistry? here are as many and as various species of compounds as in the other department. Besides, instead of fifty-four elements, we have only three or four, in most known substances. In a word, how can we, by the aid of the laws of organic chemistry, explain or class the various organized compound, which are nearly all formed of carbon, hydrogen, oxygen and sometimes nitrogen.

This is a great question in natural philosophy, a question well calculated to excite the emulation of chemists to the highest degree; once resolved, the greatest triumphs await the scientific enquirer, the mysteries of vegetation and animal life will be spread before us; we shall seize the key to all the rapid and singular modifications of matter which take place in animals and plants; and further, we shall discover the means of imitating them in our laboratories. This great question is now resolved; it only remains for us to unravel all the consequences which its solution involves. Truly it were difficult to imagine anything worthy a comparison with the simple, uniform, and beautiful laws, which have been unveiled by experiments within a few years. To produce with three or four elements, combinations more varied than those of the whole mineral kingdom, nature has taken a plan as simple as unexpected, for with these elements she has formed compounds, that possess all the properties of elementary bodies. This is the whole secret of organic chemistry.

It thus contains its elements within itself, cyanogen, fecula, benzoin, the elements of ammonia, the alcohols and analogous bodies are the true elements, and not the definite principles, carbon, hydrogen, oxygen and nitrogen, which do not exist until every trace of organization has disappeared. Mineral chemistry embraces all the bodies that result from the combinations of true elements; on the contrary, organic chemistry embraces all substances formed from compounds that act as elementary. Inorganic chemistry has simple elements, organic chemistry has compound ones, this is the whole difference. The laws of combination and of reaction are similar in both.

Perhaps we may add, by one of these foresights into the future, sometimes granted, to the philosophic vision, the least advanced of the two branches we have been discussing, may be that we consider the most so. If oxygen, sulphur and the metals be compounds, no one may foretell how and when their decomposition will be effected. If it be possible, it will require the exercise of forces that are unknown to us. In organic chemistry the difficulty is much less, and precisely the reverse, there, indeed, we know that the *elements* are compound; the whole art of the chemist consists in conducting his manipulations as to avoid their destruction which would carry them back to the state of indecomposable elements, which may be easily foreseen and prevented since it is regulated by simple laws. Thus we may nearly always recognize an organic root, and change it by combination into another, without resolving it into its elements.

Theus organic chemistry presents elements, some of which are equivalents to the metals, others analogous to oxygen, chlorine, sulphur, &c., these *roots* (*radicaux*) combine with one another or with elements, properly so called, and give rise, under the simplest laws of organic chemistry, to all organic combinations.

Mr. Dumas, here explained the design of the grand series of experiments which he and M. Leibig have conceived, for the advancement of science, and announced the plan which they propose to follow. All organic bodies are to be analyzed by them, that they have not already examined. They will submit all the analyses published, to an attentive verification, and they beg chemists willingly to subject theirs to the same rigid scrutiny. Nothing is more essential to all, than analyses upon which we may depend, and which we may use with full confidence in these systematic undertakings, which ulterior experience will often confirm, and which may serve as a starting point, for the most valuable researches. Their principle object being to characterise different bodies well, they will especially apply themselves to the discovery of the peculiar reactions of each and to determine their true atomic weight. These two philosophers have been long preparing zealous collaborators, by opening the doors of their laboratories to all young men who evinced a true love of the science.

*Archives de Medicine.*

